# Supplementary material for: GSK-3β in Dendritic Cells Exerts Opposite Functions in Regulating Cross-Priming and Memory CD8 T Cell Responses Independent of β-Catenin
Source: Vaccines (Basel). 2024 Sep 10;12(9):1037. doi: 10.3390/vaccines12091037 (PMC11436163; doi:10.3390/vaccines12091037)
Supplement: Supplementary file 1 [file vaccines-12-01037-s001.zip › vaccines-3173025-supplementary.pdf]

Supplemental Figures

Supplemental Figure S1

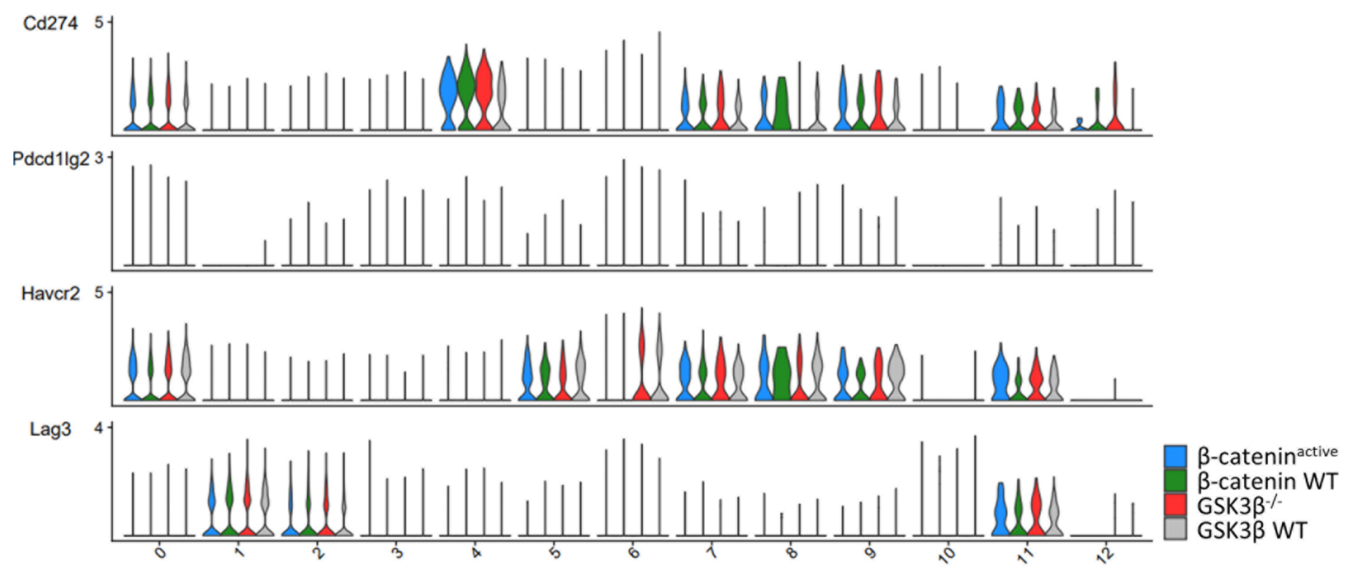

Supplemental Figure S2

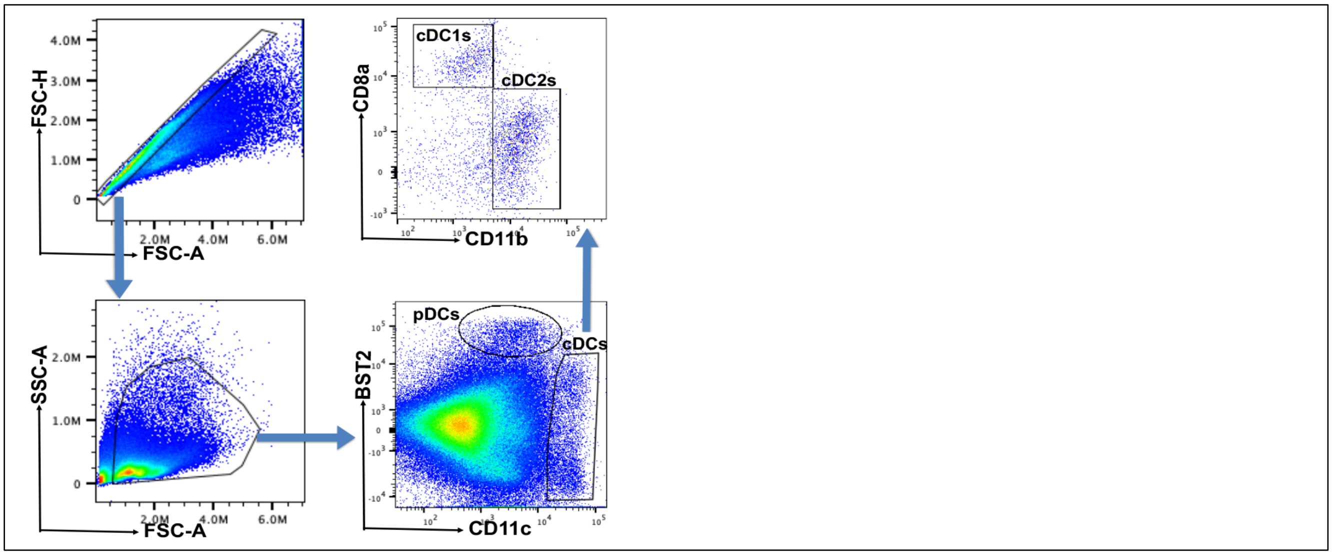

Supplemental Figure S3

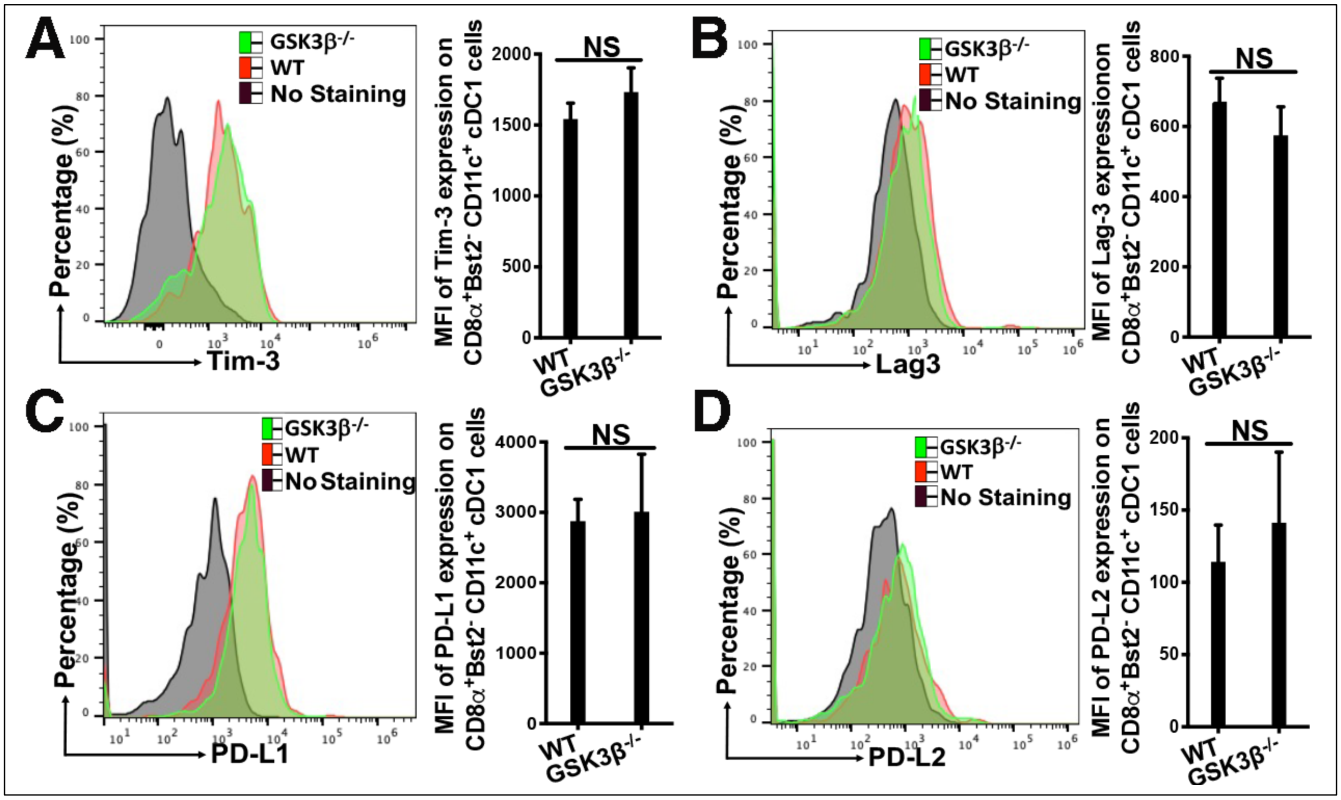

Supplemental Figure S4

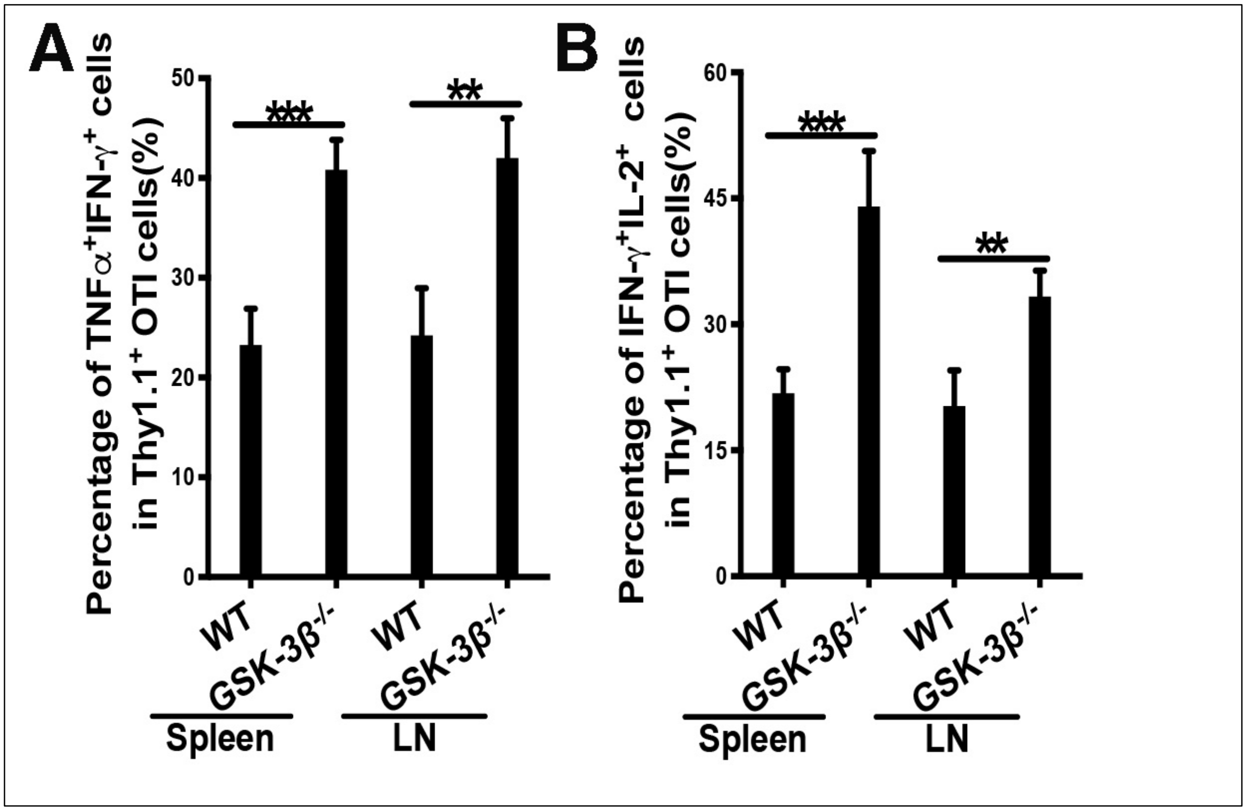

Supplemental Figure S5

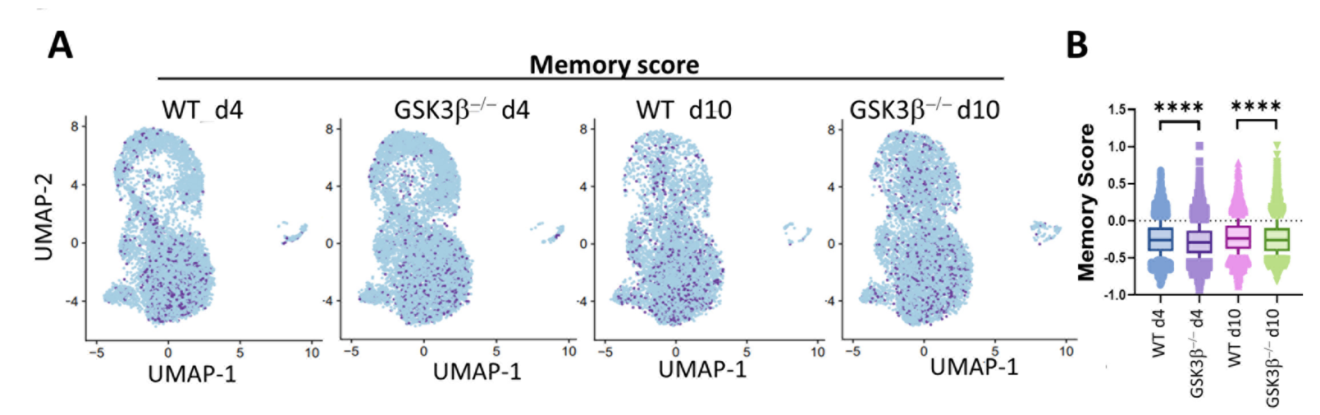

Supplemental Figure Legends

Figure S1. GSK-3β$^{-/-}$ and β-catenin ^{active} DCs exhibit different expression pattern of immune checkpoint molecules Tim-3, Lag-3, PD-L1 and PD-L2. DCs sorted from spleens of WT (GSK-3β$^{Flox/Flox}$) and CD11c-GSK-3β$^{-/-}$ mice, or from WT (β-catenin $^{Exon3/Exon3}$) and CD11c-β-catenin^{active} (CD11c-Cre β-catenin $^{Exon3/Exon3}$) were subjected to scRNA-seq as described. Violin plots depicting expression levels of inhibitory molecules Tim-3 (*Havcr2*), Lag3, PD-L1 (*Cd274*), and PD-L2 (*Pdcd1ig2*) are shown.

Figure S2. Gating strategy for splenic DCs.

Figure S3. The effects of deletion of GSK-3β on expression of inhibitory molecules Tim-3, PD-L1, PD-L2 and Lag3 in splenic cDC1s. Splenic cells from WT and CD11C-GSK-3β$^{-/-}$ mice (n=4) were stained and analyzed by flow cytometry, and the expression of inhibitory molecules Tim-3 (A), Lag3 (B), PD-L1 (C), and PD-L2 (D) on gated CD8α^{+} cDC1s as in SFigure 1 are shown on the left overlayed histograms and the right Mean Fluorescence Intensity (MFI). Student's t test, NS > 0.05. Data shown are representative of two experiments.

Figure S4. CD11c-GSK-3β$^{-/-}$ mice exhibit increased polyfunctional effectors following DC-targeted vaccination. WT and CD11c-GSK-3β$^{-/-}$ mice (n=4) were immunized with anti-DEC-205-OVA plus CpG, and cross-priming of adoptively transferred Thy1.1^{+} OTI CD8 T cells was examined at day 4 after immunization following 5 hour *in vitro* stimulation with $OTI_{257-263}$ with Brefeldin A (BFA). The percentages of IFN-γ^{+}IL-2^{+} **(A)** or IFN-γ^{+}TNFα^{+} **(B)** OTI cells in total

Thy1.1$^+$ OTI cells are shown. Data are representative of two experiments. Student's t test, $^{***}P <$ 0.001 and $^{**}P < 0.01$.

Figure S5. OTI CD8 T cells primed in CD11c-GSK-3β$^{-/-}$ mice exhibit reduced memory score. WT and CD11c-GSK-3β$^{-/-}$ mice adoptively transferred Thy1.1$^+$ OTI CD8 T cells were immunized with anti-DEC-205-OVA plus CpG. Spleen cells were harvested at day 4 or day 10 after immunization, and FACS-sorted OTI cells were subjected to scRNA-seq as described. (**A**) Expression of memory markers among the UMAP clusters. Gradient expression levels are color-coded as indicated. (**B**) Violin plot depicting the module score of gene sets associated with memory on OTI cells from either WT or CD11c-GSK-3β$^{-/-}$ mice at day 4 or day 10.
